# Towards a lightweight generic computational grid framework for biological research

**DOI:** 10.1186/1471-2105-9-407

**Published:** 2008-10-02

**Authors:** Mark D Halling-Brown, David S Moss, Adrian J Shepherd

**Affiliations:** 1 Institute of Structural and Molecular Biology, School of Crystallography, Birkbeck College, Malet Street, London, WC1E 7HX, UK

## Abstract

**Background:**

An increasing number of scientific research projects require access to large-scale computational resources. This is particularly true in the biological field, whether to facilitate the analysis of large high-throughput data sets, or to perform large numbers of complex simulations – a characteristic of the emerging field of systems biology.

**Results:**

In this paper we present a lightweight generic framework for combining disparate computational resources at multiple sites (ranging from local computers and clusters to established national Grid services). A detailed guide describing how to set up the framework is available from the following URL: .

**Conclusion:**

This approach is particularly (but not exclusively) appropriate for large-scale biology projects with multiple collaborators working at different national or international sites. The framework is relatively easy to set up, hides the complexity of Grid middleware from the user, and provides access to resources through a single, uniform interface. It has been developed as part of the European ImmunoGrid project.

## Background

In this paper we describe a Grid solution to the computational challenges arising from the ImmunoGrid project [1]. ImmunoGrid is an ambitious project that has, as its primary objective, the development of a human immune system simulator spanning multiple levels – from molecules to organs. Two main versions of the simulator are currently under development: the HIV simulator, designed to model responses to HIV-1 infection [2] and the SimTriplex simulator, designed to model vaccine schedules for the immunoprevention of mammary carcinoma in HER-2/neu transgenic mouse [3]. Both simulators are built on a single code base that is written in C and parallelised. The project has also undertaken research that requires the prediction of the location of Major Histocompatibility Complex (MHC) class I epitopes within large sets of microbial sequences (e.g. influenza strains) using pre-trained tools, and predicting the location of MHC class II epitopes using time-consuming molecular dynamics simulations.

Many of the characteristics of ImmunoGrid are shared by other biological projects: the involvement of multiple international partners (each bringing their own computational resources to the project); the need to run large numbers of computations, both large and small; and the need to provide an easy-to-use interface for a non-technical user base. From this perspective, the approach presented here can be viewed as a case study that demonstrates the relevance and effectiveness of our chosen solution to a much wider range of biological projects.

In the past, many researchers (including ourselves) have had negative experiences attempting to exploit Grid resources for scientific computation. However, significant progress has been made in recent years, notably through the development of lightweight Grid "upper middlewares" (see section below) that insulate users from the underlying access technologies. The framework presented here enables research groups to construct computational Grids that are easy to develop, modify and use.

### Requirements

With respect to our ability to access computational resources, the requirements of ImmunoGrid are as follows:

• To enable the most complex single simulations to be run, requiring access to a large cluster or supercomputer.

• To enable large sets of immune system simulations and epitope predictions to be carried out, both to explore the parameter space of the simulator and to investigate the effects of a given clinical scenario on multiple individuals.

• To support smaller-scale simulations, including runs of the ImmunoGrid educational simulators, for which standards workstations are sufficient.

As foreseen when the project name was chosen, no single partner of ImmunoGrid can guarantee access to sufficient resources to meet these requirements, so a Grid-based solution is a practical necessity.

From the end-user perspective, the requirements of our Grid solution are as follows:

• Access to the underlying computational resources should be transparent, i.e. the user should gain automatic access to the set of resources currently available to him/her without needing to be aware of their underlying organisation. In other words, the user should be insulated as far as possible from issues concerning administrative boundaries, passwords, operating systems, etc.

• Given that the potential users of our Grid-based simulators are diverse and often non-technical (with direct access by clinicians an ultimate goal), all relevant resources should be accessible via an easy-to-use interface. From this perspective, a Web interface is particularly appealing as it ensures that end-users do not need to install client software on their local machines.

### Solutions

To meet the requirements outlined above, a key priority for our computational Grid is that it maximises the range and number of resources that can be added into it, from local desktop workstations to national/international Grid services such as the UK National Grid Service [4] (NGS), the European supercomputer Grid DEISA [5] (Distributed European Infrastructure for Supercomputing Applications), and the US TeraGrid [6]. The full set of computational resources that we potentially wish to access via our Grid is listed in table 1.

**Table 1:** The middleware options that allow access to different types of computational resource.

**Resource Type**	**Middleware Access Technologies**
Individual PCs (belonging to group)	Web Service/GridSAM
Individual clusters (belonging to group)	Web Service/GridSAM
CINECA	UNICORE
NGS (UK)	Globus/GridSAM
DEISA (Europe)	UNICORE/DESHL/GridSAM
TeraGrid (US)	Globus

The desire to access a diverse range of computational resources has two practical implications. Firstly, it means we must aim to support all major existing Grid middleware and platforms. Secondly, it means that the addition of a new Grid node needs to be as easy as possible, so that individuals and organisations that have resources that can potentially be incorporated into our Grid are not deterred from doing so.

In order to meet the preceding requirements, we have sought to re-use existing solutions wherever possible. At the heart of our solution are two pieces of "upper middleware", the AHE (the Application Hosting Environment) [7,8] and DESHL (DEISA Services for the Heterogeneous management Layer) [9]. Taken together, these tools provide us with mechanisms for accessing the maximum range of resources whilst shielding us from most of the complexity associated with the underlying Grid middlewares, such as Globus [10]. Neither tool on its own is sufficient. For example, we cannot access resources at CINECA using AHE, nor local computing resources using DESHL.

Our framework also allows resources to be accessed via a third mechanism – the Web Service paradigm. A Web Service provides an Application Programming Interface (API) that enables users to seamlessly integrate a remotely-hosted service with other components of the applications they are developing. This approach is becoming increasingly popular in the field of bioinformatics, with many core services provided by organisations such as the European Bioinformatics Institute [11] (EBI) already being made available as Web Services, not just via traditional "point-and-click" Web interfaces. For ImmunoGrid, instances of our simulators can be wrapped as Web Services, deployed on a local machine, and accessed via the Grid framework described in this paper.

Given a set of available resources linked by the ImmunoGrid framework, specific resources are selected automatically by a simple job broker (by default), or manually (if so desired by the user). One important feature of our solution is that it allows for the fact that different users will have the right to run jobs on different subsets of available resources. In particular, only users who have the appropriate Grid certificate will have the right to access a given national/international Grid service (notably the NGS, DEISA and/or the TeraGrid).

The final essential ingredient of our Grid framework is its Web interface. This hides the various underlying middlewares from the user, who (given relevant permissions) can run multiple simulations on diverse computational resources at various widely-distributed sites.

## Results

The Grid infrastructure described in this paper has been used to run several contrasting applications for the ImmunoGrid project. The primary scientific aim of ImmunoGrid is to develop and validate a virtual human immune system simulator. During the development of the simulator, large numbers of runs with different versions of the simulator software have been carried out using this infrastructure. In addition, we have undertaken large-scale prediction of class I T-cell epitopes using local installations of the prediction software developed at the Center for Biological Sequence Analysis (CBS), Technical University of Denmark (DTU), Copenhagen, Denmark [12]. We are also currently developing a new method for class II T-cell epitope prediction using molecular dynamics simulations.

Here we present three representative case studies that show the amount of time saved by using our Grid-based approach to handle large-scale applications. All timings are measured in wall clock time, as this represents the most relevant measurement for end-user. The individual jobs varied significantly in their computational intensity; when run on the single Birkbeck server, times ranged from fractions of a second for a single CBS prediction to approximately two days for a molecular dynamics simulation. In these case studies, access was restricted to local resources in London (UK), Bologna (Italy) and Boston (US) that were made available by members of the ImmunoGrid Consortium. Details of these resources are given in table 2.

**Table 2:** Details of the computational resources used in the case study evaluations of the Grid infrastructure.

**Resource**	**Number of nodes**	**Specification of a single node**	**Operating System**	**RAM (Gb)**
Server, Birkbeck, London	1	4 × 2.66 GHz processors	openSUSE 10.2	4
Local cluster, Birkbeck, London	22	8 × 2.6 GHz 64-bit processors	Rocks Cluster 4.3	16
Local cluster, Dana-Farber Cancer Institute, Boston	15	2 × 3.6 GHz Xeon processors	Red Hat 3.4	2
Supercomputer, CINECA, Bologna	1280	4 × Opteron Dual Core 2.6 GHz processors	Red Hat Enterprise Linux 4	10240

### Class I T-cell epitope prediction

For this case study, 40,000 influenza protein sequences were analysed using the CBS T-cell epitope prediction software for 120 MHC alleles, giving a total of 4,800,000 jobs. We estimate (from timings for a subset of 86,552 jobs) that running the entire batch on the Birkbeck server would take approximately 155 hours. Using the Grid infrastructure presented in this paper, the total number of jobs was split equally between three resources: the Birkbeck local cluster, the Dana-Farber local cluster, and the CINECA supercomputer. In this case, the splitting of the jobs over the resources was performed by hand. Splitting of jobs over the resources can be completed by the resource broker, so long as an appropriate schedule has been implemented. Subsets of the total number of nodes were used at each resource in order to comply with fair usage guidelines. The resource usage and wall times are summarised in table 3.

**Table 3:** The distribution of jobs and wall timings for the class I epitope prediction case study.

**Resource**	**Number of nodes used**	**Number of jobs**	**Wall time**
Server, Birkbeck	1	4,800,000	155 hrs
Local cluster, Birkbeck	11	1,600,000	4 hrs 41 mins
Local cluster, Dana-Farber Cancer Institute	5	1,600,000	11 hrs 38 mins
Supercomputer, CINECA	16	1,600,000	3 hrs 39 mins

The total wall time for the Birkbeck server is an estimate.

This distribution of jobs proved highly successful, with a wall time saving of approximately 6 day (over 90%) compared to the anticipated execution time with a single machine. One important caveat, however, is that the jobs sent to the CINECA supercomputer were not held in a queue for a significant period of time. This is certainly not something that was guaranteed, and other batches of jobs submitted to CINECA have not been so fortunate. Queue length is something that can only be ascertained by directly logging onto the supercomputer at run time.

### Assessment of vaccination schedule effectiveness

For this case study, the SimTriplex simulator was used to assess the effectiveness of different vaccination schedules in the prevention of mammary carcinoma in virtual mice that represent real HER-2/neu transgenic mice [3]. We ran a total of 1,600 vaccine schedules on a population of 100 mice, giving a total of 160,000 jobs. We estimate (from timings for a subset of 100 jobs) that running the entire batch on the Birkbeck server would take approximately 203 hours.

On this occasion we were unable to use the supercomputer at CINECA as it was undergoing maintenance. We therefore submitted the jobs to the two local clusters at Dana-Farber and Birkbeck. We decided to increase the number of nodes we submitted jobs to on the Dana-Farber cluster, as it was slower than the Birkbeck cluster in the preceding case study (see Class I T-cell epitope prediction). The resource usage and wall times are summarised in table 4.

**Table 4:** The distribution of jobs and wall timings for the Simtriplex evaluation of vaccine schedules for virtual mice.

**Resource**	**Number of nodes used**	**Number of jobs**	**Wall time**
Server, Birkbeck	1	160,000	203 hrs (approx.)
Local cluster, Birkbeck	11	80,000	1 hrs 9 mins
Local cluster, Dana-Farber Cancer Institute	10	80,000	18 hrs 48 mins

The total wall time for the Birkbeck server is an estimate.

Again, we achieved a substantial saving in wall time (over 90%, equivalent to nearly 8 days) compared to the anticipated time on a single machine. It is worth pointing out, however, that the relative performance of the Dana-Farber cluster (compared to the Birkbeck cluster) was significantly worse than anticipated after the results of the first case study (see Class I T-cell epitope prediction). Indeed, had all the jobs been submitted to the Birkbeck cluster, the saving would have been significantly higher (although, given that ImmunoGrid does not have exclusive access to the Birkbeck cluster, this may not have been a practical option).

This apparent inconsistency in the performance of the Birkbeck and Dana-Farber local clusters with respect to the two case studies is not easy to explain, but neither is it entirely unexpected. Ultimately it may be attributable to a complex interplay between the CPU, memory and IO characteristics of the jobs executed, or to other factors we are unaware of (such as the possible side-effects of other jobs that happened to be running on the clusters at the same time). Consequently, we cannot guarantee that the timings would be more-or-less the same even if we ran them again with the same distribution of jobs.

### Towards class II T-cell epitope prediction

For this case study, we simulated the binding of peptides to class I MHC proteins using NAMD (NAnoscale Molecular Dynamics) ABF (Adaptive Biasing Force) software [13]. Ultimately the aim is to develop a class II prediction method, but the current lack of class II data means that we are using class I data during the development stage. We estimate (from timings for a single job) that running a batch of 120 jobs on the Birkbeck server would take approximately 5,760 hours. For this case study we sent half of the jobs to the Birkbeck cluster, and half to the supercomputer at CINECA. The resource usage and wall times are summarised in table 5.

**Table 5:** The distribution of jobs and wall timings for the prediction of class II T-cell epitope binding using ABF.

**Resource**	**Number of nodes used**	**Number of jobs**	**Wall time**
Server, Birkbeck	1	120	5760 hrs
Local cluster, Birkbeck	8	60	123 hrs 55 mins
Supercomputer, CINECA	64	60	746 hrs

The total wall time for the Birkbeck server is an estimate.

Once again we achieved a substantial saving in wall time (around 87%, equivalent to over 200 days) compared to the anticipated time on a single machine. Here the majority of the CINECA time is, in fact, queuing time.

## Discussion and conclusion

The number of scientific research projects that would benefit from having access to large-scale computational resources is increasing. With the growing prevalence of large high-throughput data sets, this is true for many sciences, but it is certainly a characteristic of systems biology research, where the need to run large numbers of computationally intensive simulations is commonplace. Many projects will have access to sufficient resources in the form of a large local cluster or (following a successful application for access time) a national/international production-quality Grid. However, many others will struggle to satisfy their demand for computational power via these routes. Moreover, to rely on a single source for access to resources is inherently risky; local clusters can fail, or require maintenance or upgrading at crucial times, and long-term access on demand is rarely available to academic users of production Grids. It is also worth emphasising that, given the demand for such resources that lengthy queuing times are commonplace.

In this paper we have presented a lightweight Grid framework that aims to provide researchers with a transparent mechanism for accessing a wide range of computational resources. It is particularly appropriate for consortia of research groups that are collaborating on a particular project, as it allows each group to contribute its own local resources to the Grid with minimal effort. We have demonstrated that the framework can be used to build a Grid capable of accessing a diverse range of computational resources (local clusters, Web Services running on a single server, supercomputers, national Grids), and have used it successfully to run a range of jobs for the ImmunoGrid project. Currently we are further extending the framework to enable job submission to PI2S2 [14], the Sicilian Grid using gLite [15] middleware [16].

The hallmarks of our framework are its flexibility, ease of installation, and ease of use. This comes at a certain cost, however. Currently it is not feasible to rapidly develop a new Grid infrastructure capable of integrating such a diverse range of resources that is of production quality. Production Grids typically require services to be constantly monitored, sophisticated schemes for handling errors, and the provision of dedicated user support [17]. Indeed, it is worth noting that even national Grid services are typically rather limited in this respect. For example, users of the NGS need to manually interact with individual Grid nodes in order to ascertain which resources have the shortest queues. The time required to develop such a Grid makes it an impractical proposition for all but the largest and longest-running projects, and no off-the-shelf solution is currently available. (As noted in the section below, we have deliberately designed the framework in a modular way, so that when a suitable meta-broker becomes available, we will be able to utilise it.) Nevertheless, notwithstanding the non-optimal deployment of resources, we believe the Grid framework presented here represents a reasonable compromise.

## Methods

In this paper we are primarily concerned with describing the functionality of the Grid infrastructure that has been developed and used by the ImmunoGrid project, with particular emphasis on its flexibility, ease of deployment, and ease of use. Hence the focus here is on the upper layers of our infrastructure. This paper describes the provision of a generic interface to DESHL, the AHE, and Web Services. These solutions were selected on the basis of the combined coverage they provide in terms of access to computational resources (as summarised in table 1), and their relative ease of deployment and use. As we will show, the effort required to integrate these solutions within a single, coherent infrastructure is comparatively modest. Most of the tasks described do not require a specialist developer as a detailed guide is provided. However, when customisation of the interface or alterations to the resource broker or other aspects of the grid infrastructure are required, a technically competent user will be necessary.

**Figure 1:**
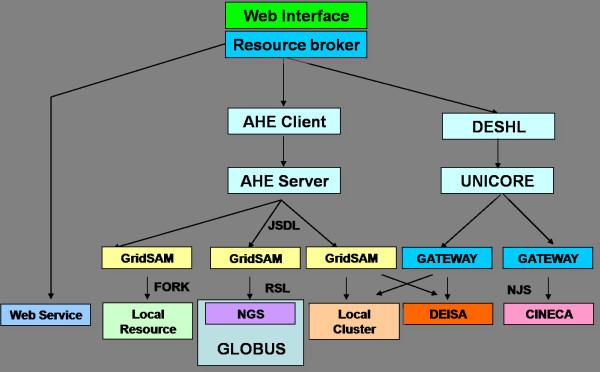
**A schematic overview of the Grid infrastructure described in this paper**. Abbreviations are as follows: JSDL = Job Submission Description Language; RSL = Resource Specification Language; NJS = Network Job Scheduler.

A schematic overview of our Grid infrastructure is presented in figure 1. At the top level it comprises a single web-based interface coupled to a resource broker/job launcher. The broker/launcher accesses DESHL and the AHE via middleware-specific scripts, and Web Services via standard SOAP protocols. The main benefit of this approach is that it allows us to launch jobs simultaneously on different national/international Grid services, on local computational resources and as Web Services through a single, unified interface. Using this approach, we effectively hide the complexity and diversity of the underlying middlewares and resources from the end user.

### Upper middleware

Arguably the most important ingredient in our framework is the role played by "upper middleware" (AHE and DESHL), as it hides much of the complexity of the Grid both from those developing a new Grid infrastructure and from the users of that infrastructure. Without upper middleware, the whole enterprise would be prohibitively complicated and time-consuming for most scientific institutions or consortiums to undertake. AHE and DESHL play a key role in the deployment of software to different computational resources and in the management of jobs. Both AHE and DESHL provide a command-line interface via which a job can be launched, its progress monitored, and its output (both intermediate and final) retrieved. There are, however, some important differences.

The Application Hosting Environment (AHE) is a lightweight environment designed to run unmodified applications on diverse, distributed Grid resources. An explicit design goal of AHE is to hide the underlying complexity (of the Grid middleware, of the host environment and of how executables are set up) from the end user. Currently this is achieved using GridSAM [18] (Grid Job Submission and Monitoring Web Service), but a UNICORE (Uniform Interface to Computing Resources [19]) plugin recently been developed and has been used by the Coveney group [20]. The UNICORE plugin is not available as part of the AHE package. After the initial deployment of AHE, a simple Job Submission Description Language (JSDL) file must be produced for each combination of software and resource that the AHE will have access to. This is the only manual intervention required, and it need only be done once for each software/resource combination. Thereafter, the AHE provides a list of available resources upon which the software has been installed. Jobs can then be run by simply selecting resources from the list (see Resource brokering and job launching); there is no need to access any resources directly. The AHE is easy to install as part of the OMII [21] stack.

DESHL is somewhat less flexible than the AHE, but it does provide essential mechanisms for accessing European supercomputers via UNICORE. In the context of the ImmunoGrid project, such resources are available via DEISA and at CINECA (a member of the ImmunoGrid Consortium). Setting up access to a supercomputer via DESHL is somewhat less transparent than adding a resource using the AHE, as scripts need to be written that manage access to a named user space.

### Accessing local resource

There are two ways that a local resource can be accessed via our system – using the AHE, or as a Web Service. The fundamental difference between these two approaches is that a given resource is made available for any application using the AHE route, whereas the Web Services approach makes a specific application available. The practical differences between these two approaches are relatively minor.

In order to access a new local resource using the AHE, an Apache Tomcat [22] server needs to be installed on the local machine together with an instance of GridSAM (an Open Source job submission and monitoring Web Service). These are automatically installed and configured (without recourse to special administrative rights) when the OMII stack is installed on the machine. The final step is simply to edit the configuration of the GridSAM instance so that it points to the locally installed software that we wish to use.

Providing access to a local resource using Web Services is slightly more complicated. It can be achieved either by 'wrapping' the software in a simple Web Service shell or by pointing a Web Service execution at the local software. In either case, this involves writing some code, such as a Web Service Definition Language (WSDL) file. An application server or Web server is required to host the Web Service.

Both of these approaches to the incorporation of local resources into a Grid are documented in detail on the portal website [23].

### Security

Both UNICORE and AHE handle security and authorisation using X.509 digital certificates [24]. This largely enables us to manage the security of our Grid using a single, uniform approach. Users who have their own Grid certificates for accessing NGS and DEISA resources are able to upload them, thereby gaining access to those resources. However, we anticipate that the majority of end users will not have their own certificates. To allow such users access to local Grid resources, a self-signed certificate can be generated for the portal or each user. This self-signed certificate will provide access to local Grid resources only.

Both UNICORE and AHE (together with most X.509 certificate authorised (CA) middlewares) require that the certificate is presented on a MyProxy server [25] (this includes the self-signed certificates). This ensures that the certificate's password need only be entered once during the submission process. To enable certificates to be deployed in this manner, the Web interface to our system has a link to the Java Web Start [26] JNLP [27] (Java Network Launching Protocol) MyProxy Upload Tool. Although this is not a fully automated solution (as it requires the user to manually enter the location of the certificate as well as enter the password), it is currently the most reliable. The self-signed certificates are handled in exactly the same manner as the CA certificates so provide the same security features.

### Resource brokering and job launching

Currently our infrastructure uses a simple PHP script to handle resource brokering and job launching. Jobs are allocated to resources according to whether the user has a Grid certificate, and by taking into account the anticipated job length compared to any limits imposed by specific resources. For example, when the job involves running the ImmunoGrid simulator, specific settings within the simulator configuration file (such as the maximum number of iterations it will run for, and the length of the bit-string used to represent molecular interactions) are used to estimate job length. From the list of resources deemed to be both available and appropriate for running a given job, specific resources are allocated at random. Alternatively, the end user may select which of the appropriate resources they wish to uses for running a given batch of jobs.

Although this approach is sufficient for our current requirements, a more sophisticated resource broker that seeks to optimise total execution time and ensure fairness within the context of agreed policies on resource usage will be appropriate for many applications. A wide range of approaches are possible [28]. Unfortunately, deploying an existing Grid resource broker within our framework is currently problematic, as each broker supports only a subset of possible middlewares. However, this situation may change in the future, given the present interest in Grid resource meta-brokering [29].

Jobs are launched by a simple job launcher. This executes the appropriate launch command for a given job and resource (this is different for the AHE, DESHL, and Web Services). The launcher also records the details of the job both on the server's filesystem and in a local database, and executes the appropriate command line script corresponding to the resource that is selected. The state of the job is stored in the local database along with any information required to uniquely identify that job. This allows the appropriate scripts to be called when the user requests the job state to be refreshed.

Assuming a given batch job requires a large number of individual parameter files to be created (e.g. a single parameter file for each job containing the unique parameter settings for that job), these are generated using a Perl script. Where appropriate, it would be relatively straightforward to provide a Web interface that would enable end-users to generate multiple parameter files without having to run the Perl scripts directly, but the design of such an interface is application specific.

### Web Interface

**Figure 2:**
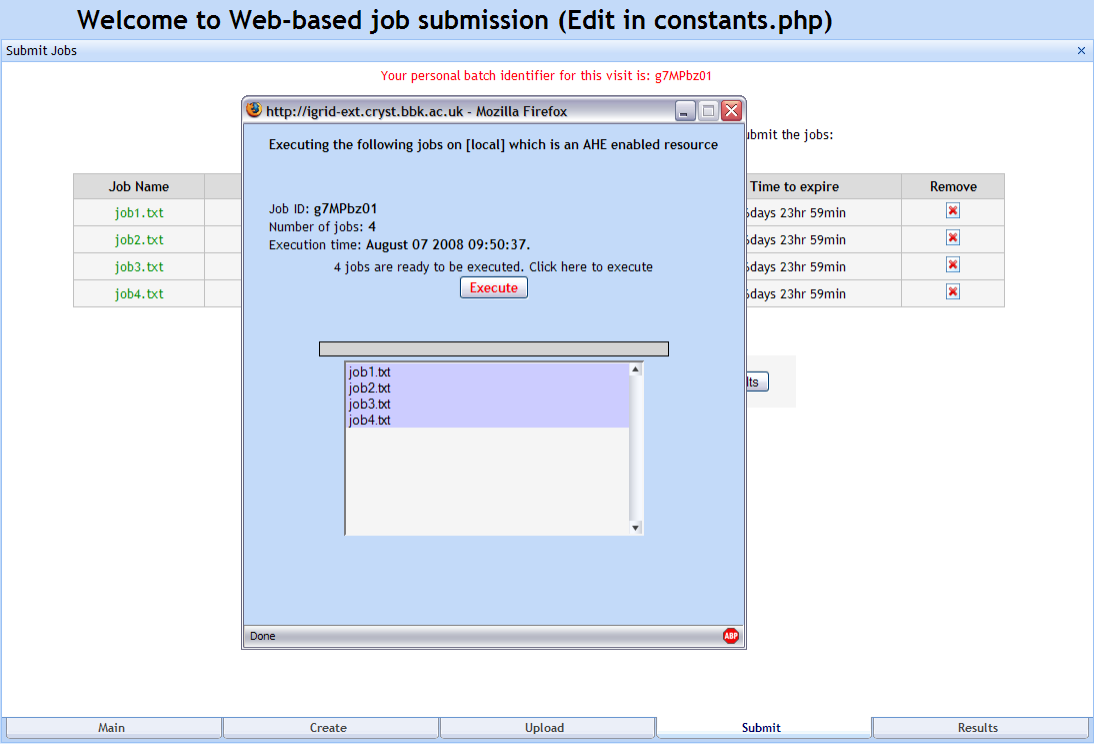
**A screenshot of the job submission process.** Jobs are being submitted to a local AHE resource.

The Web interface to our infrastructure provides the end user with simple mechanisms for uploading, launching and monitoring the progress of jobs, as well as for retrieving and displaying results. The interface comprises PHP Web pages, with AJAX and DHTML used to give them a modern look and feel. Figure 2 shows a screen shot of the interface.

The interface is loosely coupled to the resource broker/job launcher and has been developed in a modular way to facilitate it being adapted to handle new applications. In this respect, the module that handles the output files generated by a given batch of jobs is a key issue, as the appropriate behaviour for this module is application specific. For our ImmunoGrid simulator application, we have developed an interface using the JPGraph [30] object-oriented graph-creation library that enables the user to visualise how various simulator parameters (e.g. the levels of antigen, T-cells and B-cells in the system) varied over the run time of a given simulation. However, for the public downloadable version of our framework, the default behaviour is to provide the user with access to a tar file containing all the output files generated by a given job.

**Figure 3:**
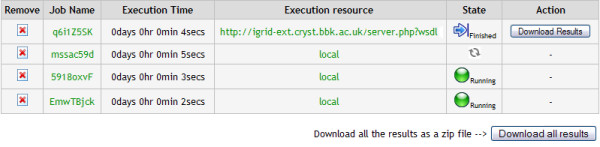
A screenshot of the different execution states of some example jobs.

It is possible that submitted jobs may fail or give errors. The grid middlewares utilised in this paper report when a job has failed. The user is informed about the state of their jobs when viewing the results section of a specific job. This section provides a review of the jobs submitted the date they were submitted, the time to expire and the state that each job is in. The state of the jobs is automatically polled periodically. The states that a job can be are *executed*, *running*, *finished *or *error*. An example of some jobs in various states can be seen in figure 3.

The instructions for implementing the web interface are contained on the portal guide site: . There is also a link to a demo of the web interface available from the portal guide.

## Authors' contributions

All authors conceived of the study, MDHB was responsible for its design and implementation and helped to draft the manuscript. DSM and AJS conceived of the study and were involved in its design and helped to draft the manuscript. All authors read and approved the final manuscript.
